# Whole-transcriptome analysis of differentially expressed genes in the mutant and normal capitula of *Chrysanthemum morifolium*

**DOI:** 10.1186/s12863-021-00959-2

**Published:** 2021-01-25

**Authors:** Hua Liu, Chang Luo, Dongliang Chen, Yaqin Wang, Shuang Guo, Xiaoxi Chen, Jingyi Bai, Mingyuan Li, Xinlei Huang, Xi Cheng, Conglin Huang

**Affiliations:** 1 Beijing Agro-Biotechnology Research Center, Beijing Academy of Agriculture and Forestry Sciences, Beijing Engineering Research Center of Functional Floriculture, Beijing, Key Laboratory of Agricultural Genetic Resources and Biotechnology, Beijing, 100097 China; 2 Beijing Vegetable Research Center, Beijing Academy of Agriculture and Forestry Sciences, Beijing, 100097 China

**Keywords:** *Chrysanthemum morifolium*, Ray florets, Pistils, Flower development, Mutant capitula, Anthocyanin biosynthesis, Whole-transcriptome analysis, Differentially expressed genes

## Abstract

**Background:**

*Chrysanthemum morifolium* is one of the most economically important and popular floricultural crops in the family Asteraceae. Chrysanthemum flowers vary considerably in terms of colors and shapes. However, the molecular mechanism controlling the development of chrysanthemum floral colors and shapes remains an enigma. We analyzed a cut-flower chrysanthemum variety that produces normal capitula composed of ray florets with normally developed pistils and purple corollas and mutant capitula comprising ray florets with green corollas and vegetative buds instead of pistils.

**Results:**

We conducted a whole-transcriptome analysis of the differentially expressed genes (DEGs) in the mutant and normal capitula using third-generation and second-generation sequencing techniques. We identified the DEGs between the mutant and normal capitula to reveal important regulators underlying the differential development. Many transcription factors and genes related to the photoperiod and GA pathways, floral organ identity, and the anthocyanin biosynthesis pathway were differentially expressed between the normal and mutant capitula. A qualitative analysis of the pigments in the florets of normal and mutant capitula indicated anthocyanins were synthesized and accumulated in the florets of normal capitula, but not in the florets of mutant capitula. These results provide clues regarding the molecular basis of the replacement of *Chrysanthemum morifolium* ray florets with normally developed pistils and purple corollas with mutant ray florets with green corollas and vegetative buds. Additionally, the study findings will help to elucidate the molecular mechanisms underlying floral organ development and contribute to the development of techniques for studying the regulation of flower shape and color, which may enhance chrysanthemum breeding.

**Conclusions:**

The whole-transcriptome analysis of DEGs in mutant and normal *C. morifolium* capitula described herein indicates the anthocyanin deficiency of the mutant capitula may be related to the mutation that replaces ray floret pistils with vegetative buds. Moreover, pistils may be required for the anthocyanin biosynthesis in the corollas of chrysanthemum ray florets.

**Supplementary Information:**

The online version contains supplementary material available at 10.1186/s12863-021-00959-2.

## Background

*Chrysanthemum morifolium* is one of the most economically important and popular floricultural crops in the family Asteraceae, and ranks second in the cut-flower industry after rose [1]. The head-like inflorescence (capitulum), which resembles a single large flower, is the main ornamental part of *C. morifolium* and is considered to be important for the evolutionary success of Asteraceae species [2]. The typical chrysanthemum capitulum is formed by two morphologically distinct florets, the marginal ray florets and the central disk florets. Ray florets have ligulate and zygomorphic colorful corollas (petals) and aborted stamens, which attract pollinators. The disk florets have radially symmetrical colorless corollas, and their fertile pollen grains are hermaphroditic and used for reproduction (Additional file 1). The diverse flower colors and shapes are the most visible results of floral evolution and have influenced the desirability of certain flowers to humans [3].

Flowering, which is a key developmental process in the plant life cycle, is a very complex process controlled by endogenous factors and environmental cues. More specifically, floral development comprises the following three phases: flowering determination, flower evocation, and floral organ development [4]. Regarding *Arabidopsis thaliana*, there has been substantial progress toward elucidating the molecular mechanisms underlying floral development [5, 6]. The ABCDE models have revealed that A-class and E-class genes specify sepal identity; A-class, B-class, and E-class genes specify petal identity; B-class, C-class, and E-class genes determine stamen identity; C-class and E-class genes determine carpel/gynoecium organ identity; C-, E- and D-class genes specify ovule identity and differentiation [7]. With the notable exception of A-class genes, all of these genes belong to the MADS-box family of transcription factors, including the *AP1*, *AP3*, *PI*, *AG*, and *SEP* genes.

The diversity in plant colors, especially among flower petals, has enabled plants to continually develop new showy traits and prosper throughout millions of years of evolution. Anthocyanins and carotenoids are the two major groups of pigments generated in plant petals. Anthocyanins accumulate in the vacuoles of petal epidermal cells and confer orange-to-violet colors in flowers [8]. In addition to attracting pollinators, anthocyanins also protect plants from UV irradiation [9]. Anthocyanins provide chrysanthemum ray florets with bright colors to attract pollinators, thereby increasing the cross pollination rate of different species or varieties and promoting the development of cultivar groups with highly variable flower types*.* Anthocyanins enhance the ornamental value of chrysanthemums, and many cut-flower and pot-flower varieties with bright colors are produced annually to satisfy market demands. Clarifying the mechanism regulating anthocyanin biosynthesis may enable researchers and breeders to produce novel chrysanthemum varieties with new flower colors.

In chrysanthemums, a few floral development regulatory genes have been isolated such as MADS-box, *TCP*, and *WUS*-like genes [10–13]. Some important functional genes and transcription factors involved in the anthocyanin biosynthesis pathway have also been characterized, including *ANS*, *F3′H*, *F3H*, and *MYB*-like genes [13–16]. However, chrysanthemum capitula contain two morphologically distinct florets. Moreover, long-term breeding efforts have resulted in diverse flower shapes and colors. The mechanism underlying the evolution and development of chrysanthemum flowers is complex and remains relatively uncharacterized.

The development of RNA sequencing (RNA-seq) technology has greatly improved transcriptomic analyses of chrysanthemums [1]. However, the reads of second-generation high-throughput sequencing platforms are much shorter than the typical length of a eukaryotic mRNA. Additionally, the differences in transcript abundance and the presence of different unigenes make the assembly of transcriptomes from short reads extremely challenging [17]. Despite these problems, Hirakawa et al. used the Illumina sequencing platform for the de novo assembly of the whole *Chrysanthemum seticuspe* genome and Chi Song et al. sequenced the diploid *Chrysanthemum nankingense* genome using the Oxford Nanopore long-read technology [18, 19]. Unfortunately, no more than 40% of the transcriptome sequencing reads from *C. morifolium* can be mapped to these two genome sequences, probably because of the extreme variation in chromosome ploidy and biological characteristics. Third-generation sequencing technology has dramatically increased the length of sequencing reads, enabling the precise localization and sequencing of repetitive regions and unigenes with a single read.

We recently obtained a mutant plant of the cut-flower chrysanthemum variety *C. morifolium* ‘ZY’ with both normal and mutant capitula. The normal capitula were composed of many rounds of ray florets with purple corollas and normally developed pistils, whereas in the mutant capitula, the ray floret corollas were green and the pistils were replaced by vegetative buds. In this study, we applied the Pacific Biosciences (PacBio) single-molecule long-read sequencing technology to analyze a mixed sample of normal and mutant flowers, leaves, stems, and roots from ‘ZY’ plants. On the basis of the results, transcripts were sequenced and the mutant and normal capitula were examined using second-generation sequencing and RNA-seq technology. Thus, we combined third-generation and second-generation sequencing techniques to generate a more complete *C. morifolium* transcriptome.

Transcriptome sequencing and analysis revealed differentially expressed genes (DEGs) between the mutant and normal capitula, some of which may encode important regulators controlling the differential development. Many transcription factors and genes related to the photoperiod and gibberellin (GA) pathways, floral organ identity, and the anthocyanin biosynthesis pathway were differentially expressed between the normal and mutant capitula. These results may be useful for clarifying the molecular mechanisms underlying the phenotypic differences between ray florets with normally developed pistils and purple corollas and mutant ray florets with green corollas and vegetative buds in *C. morifolium*. Moreover, the data presented herein may elucidate the molecular basis of floral organ development, with implications for the development of techniques suitable for studying the regulation of flower shape and color and the breeding and molecular characterization of chrysanthemum.

## Results

### Sequencing and assembly

**Fig. 1:**
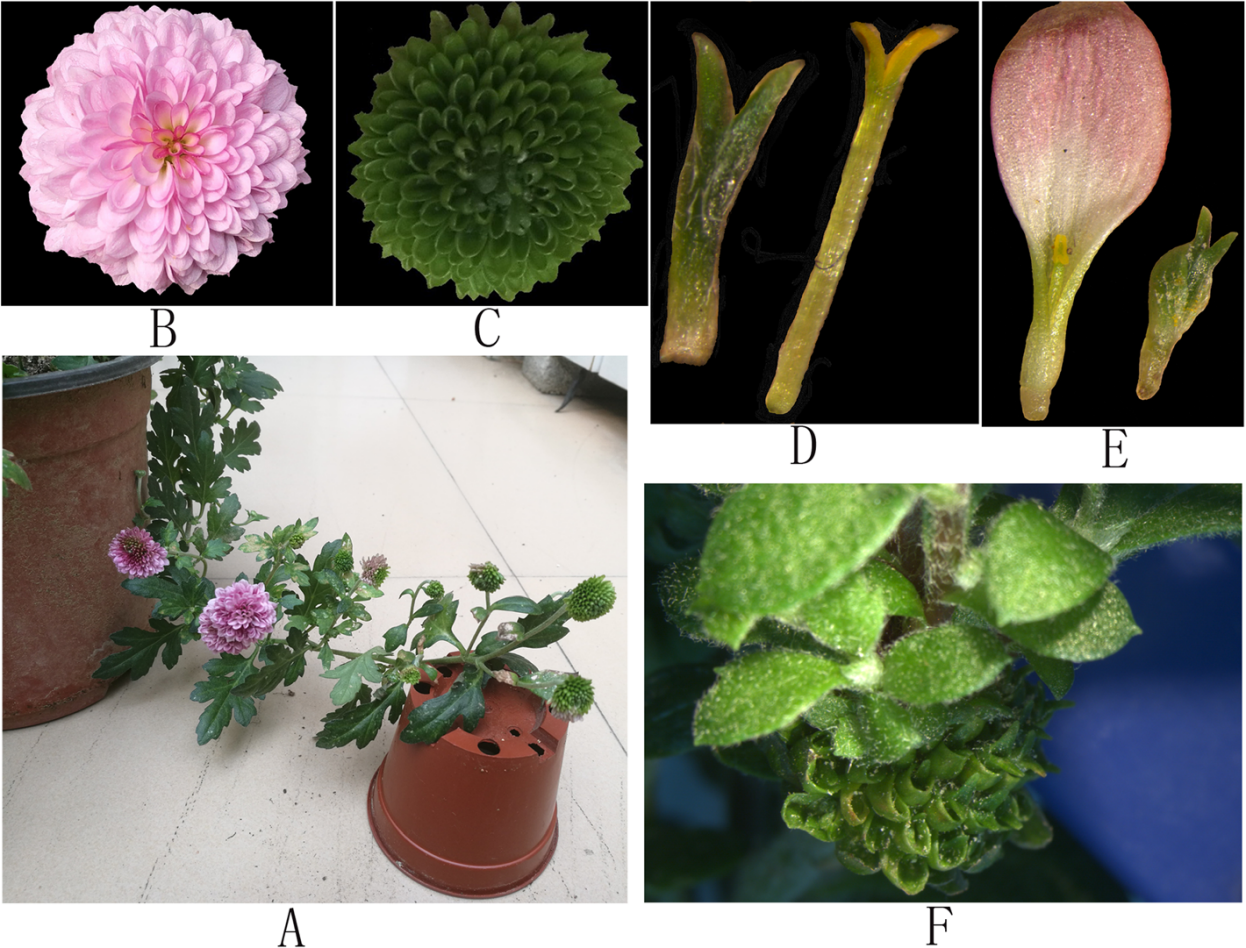
Mutant Chrysanthemum morifolium ‘ZY’ plant. (**a**) Mutant and normal capitula. (**b**) Normal capitulum. (**c**) Mutant capitulum. (**d**) Vegetative buds in the mutant capitulum (left) and pistils in the normal capitulum (right). (**e**) Normal ray florets (left) and mutant ray florets (right). (F) New shoots from the mutant capitulum

The *C. morifolium* ‘ZY’ plants analyzed in this study produced both normal and mutant capitula (Fig. 1). The normal capitula were composed of many rounds of ray florets with purple corollas and normally developed pistils. In contrast, the mutant capitula consisted of many rounds of mutant ray florets with green corollas as well as vegetative buds instead of pistils. We analyzed *C. morifolium* ‘ZY’ normal and mutant capitula, leaves, stems, and roots using PacBio sequencing, after which the normal and mutant capitula were separately analyzed using Illumina paired-end sequencing technology. The resulting sequences were assembled into 130,097 unigenes with an N50 of 3013 bp and an average length of 2510 bp (Table 1).

**Table 1:** De novo assembly results

Unigene	N50 (bp)	Max length (bp)	Min length (bp)	Average length (bp)	Total assembled bases	GC%	Annotation counts	Annotation ratio
130,097	3013	14,273	57	2510	494,377,327	40.06	118,589	90.73%

### Gene annotation and functional classification

**Fig. 2:**
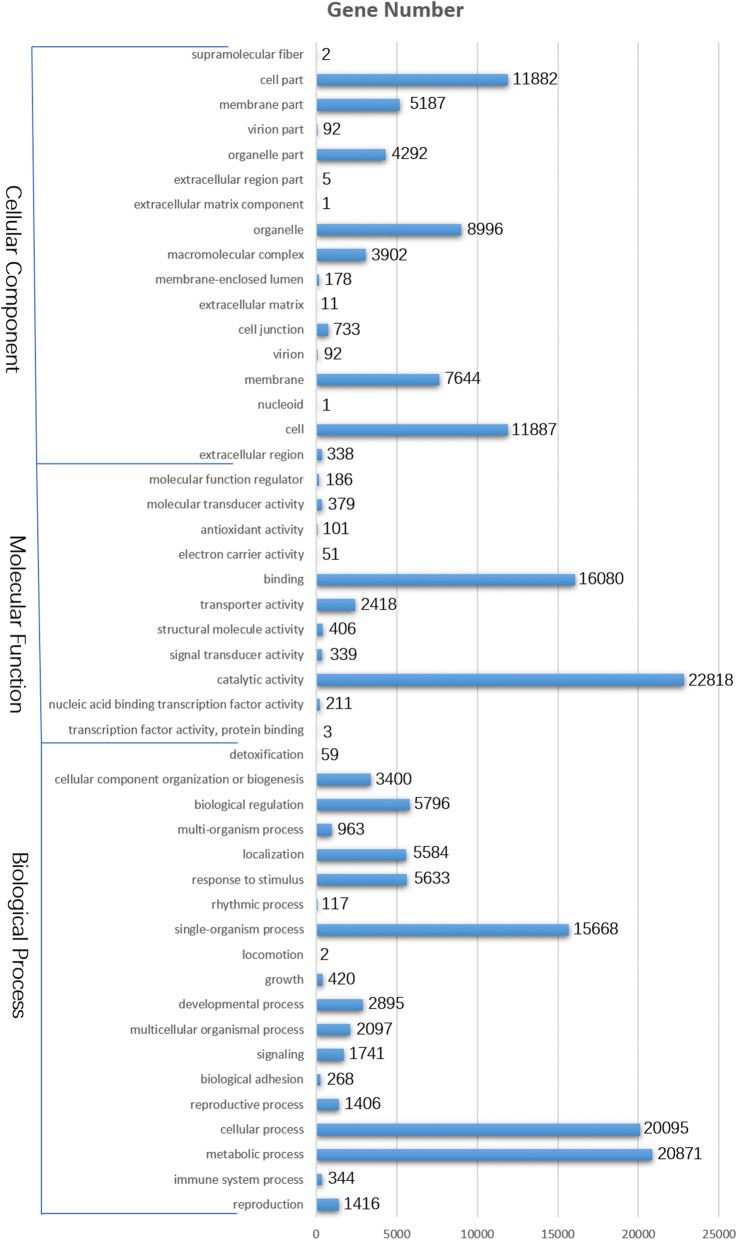
Histogram of Gene Ontology classifications. The genes are divided in three main categories: biological process, cellular component, and molecular function. The y-axis on the left side indicates the percentage of genes in a category, whereas the y-axis on the right side presents the number of genes

A total of 118,589 unigenes were annotated following a BLAST search of four databases [non-redundant (nr) protein database, Swiss-Prot, EuKaryotic Orthologous Groups (KOG), and Kyoto Encyclopedia of Genes and Genomes (KEGG)], leaving 11,508 (8.85%) unannotated unigenes. A total of 118,043, 101,048, 87,630, and 54,245 unigenes were annotated on the basis of searches of the nr, Swiss-Prot, KOG, and KEGG databases, respectively. Moreover, the Gene Ontology (GO) database was used for the functional annotation and analysis of genes, which were divided into the following three main categories: molecular function, cellular component, and biological process. Specifically, 36,144 unigenes were classified into 47 functional categories, including 19, 17, and 11 in the biological process, cellular component, and molecular function categories, respectively. The predominant biological process, molecular function, and cellular component GO terms among the genes were ‘metabolic process’ (20,871), ‘catalytic activity’ (22,818), and ‘cell’ (11,887), respectively. This implied that numerous metabolic activities were activated during the development of chrysanthemum capitula in a process regulated by the combined effects of the proteins encoded by these diverse genes. Additionally, a substantial proportion of the genes were annotated with the ‘cellular process’, ‘binding’, and ‘cell part’ GO terms, whereas ‘locomotion’, ‘transcription factor activity, protein binding’, and ‘extracellular matrix component’ were relatively uncommon GO terms (Fig. 2).

**Fig. 3:**
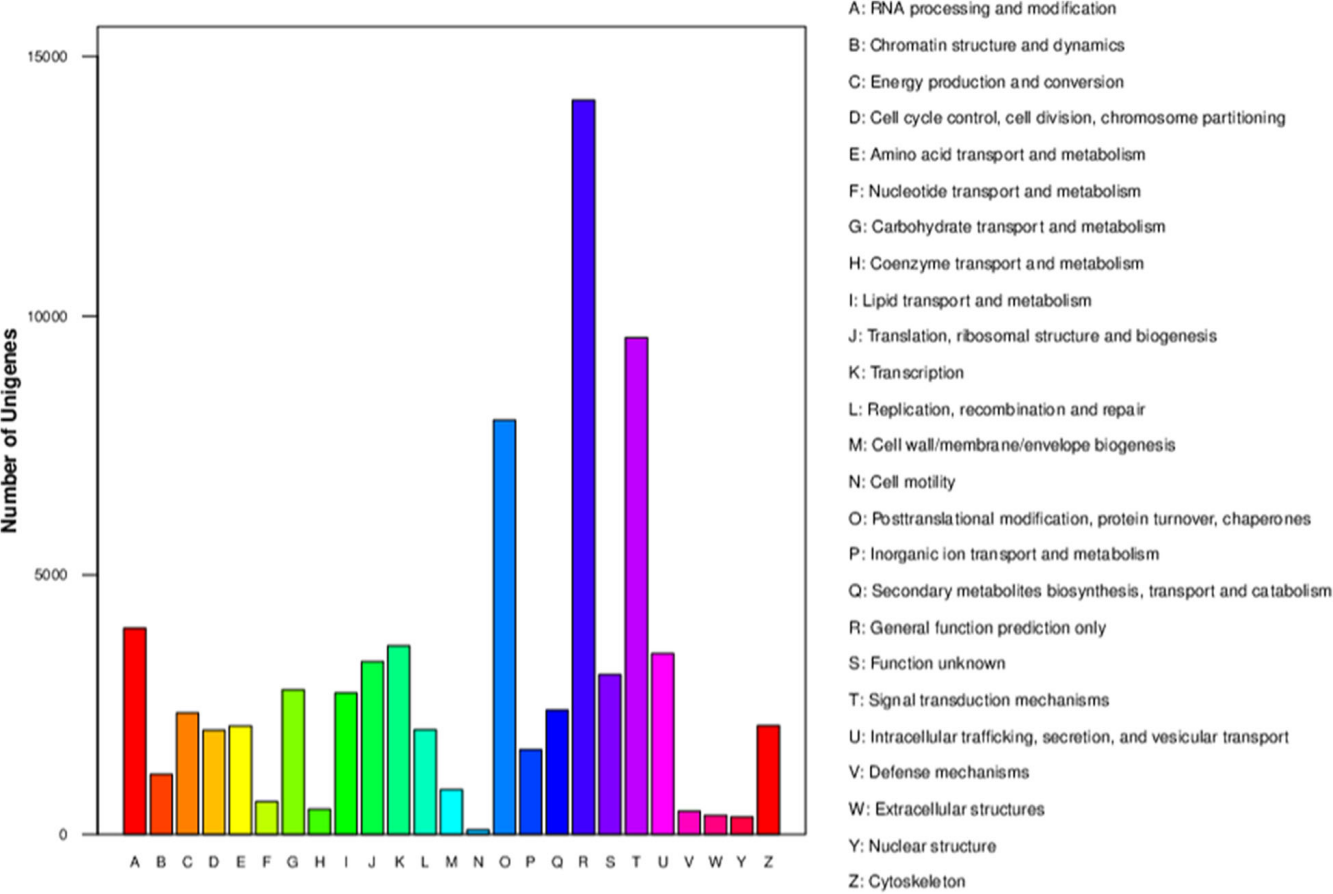
EuKaryotic Orthologous Groups (KOG) classifications in chrysanthemum. A total of 87,630 sequences classified in 25 KOG categories are presented

The KOG database is usually used to identify orthologous and paralogous proteins. Additionally, JGI-predicted genes may be identified according to KOG classifications or IDs. The annotated sequences were used as queries to screen the KOG database to assess the completeness of our transcriptome library and the reliability of our annotation process. Of 118,043 nr hits, 87,630 sequences were assigned KOG classifications. Among the 25 KOG categories, the cluster for ‘general function prediction only’ (28,904, 32.98%) represented the largest group, followed by ‘signal transduction mechanisms’ (23,030, 26.68%) and ‘posttranslational modification, protein turnover, chaperones’ (19,436, 22.18%). Conversely, the ‘defense mechanisms’ (673, 0.77%), ‘extracellular structures’ (454, 0.52%), and ‘cell motility’ (170, 0.19%) clusters were the smallest groups (Fig. 3).

To further evaluate the chrysanthemum transcriptome, all unigenes were aligned with the sequences in the KEGG database using the BLASTx algorithm (E-value < 10^− 5^). As a collection of manually drawn pathway maps, KEGG pathways present the networks of molecular interactions in cells and particular organisms. Of the 118,043 unigenes, 54,245 had significant matches with at least one KEGG pathway in the database and were assigned to 133 KEGG pathways in total (Table 2). The most represented pathways were ‘metabolic pathways’ (12,473 members) and ‘biosynthesis of secondary metabolites’ (6980 members), followed by ‘biosynthesis of antibiotics’ (3268 members), ‘microbial metabolism in diverse environments’ (2777 members), and ‘carbon metabolism’ (2089 members). Additionally, 1339 unigenes were associated with the ‘plant hormone signal transduction’ pathway.

**Table 2:** Enriched KEGG pathways among chrysanthemum unigenes

KEGG Categories	Unigene number	Rotio of no.	Pathway ID
Metabolic pathways	12,473	40.16%	ko01100
Biosynthesis of secondary metabolites	6980	22.47%	ko01110
Biosynthesis of antibiotics	3268	10.52%	ko01130
Microbial metabolism in diverse environments	2777	8.94%	ko01120
Carbon metabolism	2089	6.73%	ko01200
Protein processing in endoplasmic reticulum	1858	5.98%	ko04141
Biosynthesis of amino acids	1766	5.69%	ko01230
Spliceosome	1725	5.55%	ko03040
Endocytosis	1546	4.98%	ko04144
Starch and sucrose metabolism	1511	4.86%	ko00500
RNA transport	1350	4.35%	ko03013
Plant hormone signal transduction	1339	4.31%	ko04075
Ubiquitin mediated proteolysis	1205	3.88%	ko04120
Plant-pathogen interaction	1202	3.87%	ko04626
mRNA surveillance pathway	1085	3.49%	ko03015
Purine metabolism	1080	3.48%	ko00230
RNA degradation	1045	3.36%	ko03018
Ribosome	1016	3.27%	ko03010
Oxidative phosphorylation	1013	3.26%	ko00190
Aminoacyl-tRNA biosynthesis	933	3.00%	ko00970
Amino sugar and nucleotide sugar metabolism	898	2.89%	ko00520
Glycolysis / Gluconeogenesis	811	2.61%	ko00010
Pyruvate metabolism	799	2.57%	ko00620
Glycerophospholipid metabolism	793	2.55%	ko00564
Pyrimidine metabolism	790	2.54%	ko00240
Glyoxylate and dicarboxylate metabolism	781	2.51%	ko00630
Peroxisome	711	2.29%	ko04146
Fatty acid metabolism	708	2.28%	ko01212
Phagosome	699	2.25%	ko04145
alpha-Linolenic acid metabolism	678	2.18%	ko00592
Cysteine and methionine metabolism	667	2.15%	ko00270
Carbon fixation in photosynthetic organisms	614	1.98%	ko00710
Glycine, serine and threonine metabolism	583	1.88%	ko00260
Phosphatidylinositol signaling system	556	1.79%	ko04070
Phenylpropanoid biosynthesis	525	1.69%	ko00940

### Alternatively spliced unigenes

**Fig. 4:**
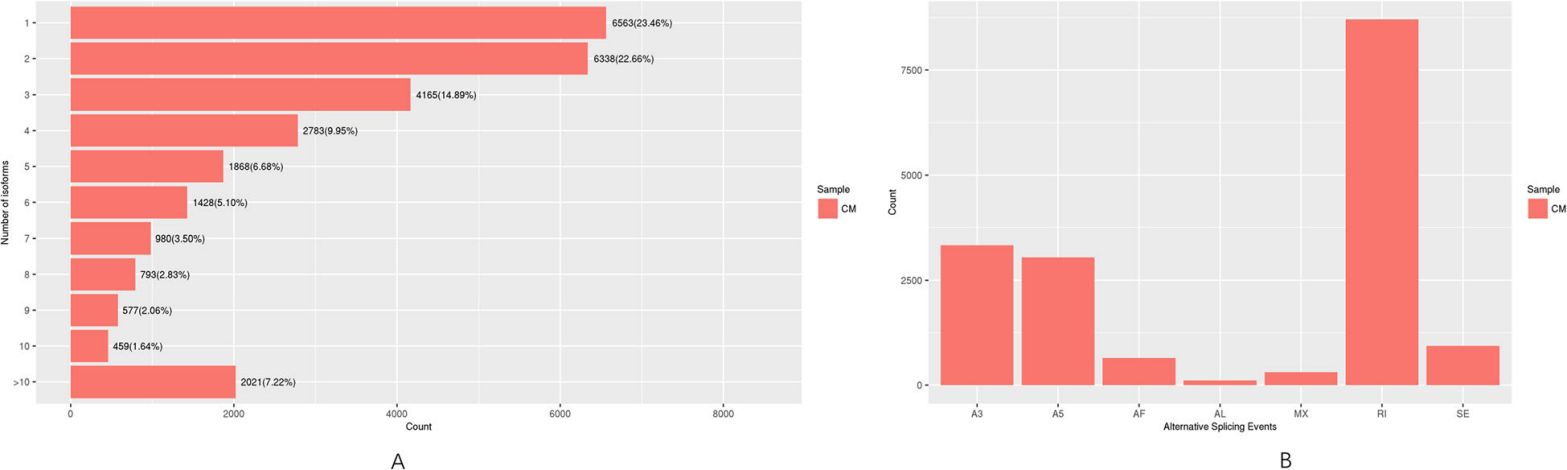
Alternatively spliced genes. (A) Alternatively spliced unigenes. (B) Alternative splicing types

The long PacBio sequencing reads can provide extensive information about alternative splicing. In this study, 27,975 unigenes had two or more alternatively spliced isoforms, 15,074 had three or more distinct isoforms, and 10,909 had four or more distinct isoforms (Fig. 4a). Seven alternative splicing types were identified based on a SUPPA analysis, including exon skipping (938, 5.5%), alternative 5′ splice site (3044, 17.8%), alternative 3′ splice site (3336, 19.5%), mutually exclusive exon (305, 1.8%), retained intron (8705 51%), alternative first exon (646, 3.8%), and alternative last exon (108, 0.6%). Therefore, retained intron, alternative 3′ splice site, and alternative 5′ splice site were the main alternative splicing types (Fig. 4b).

### Comparison of the transcriptomes of normal and mutant capitula

Unigenes common to normal and mutant capitula.

**Fig. 5:**
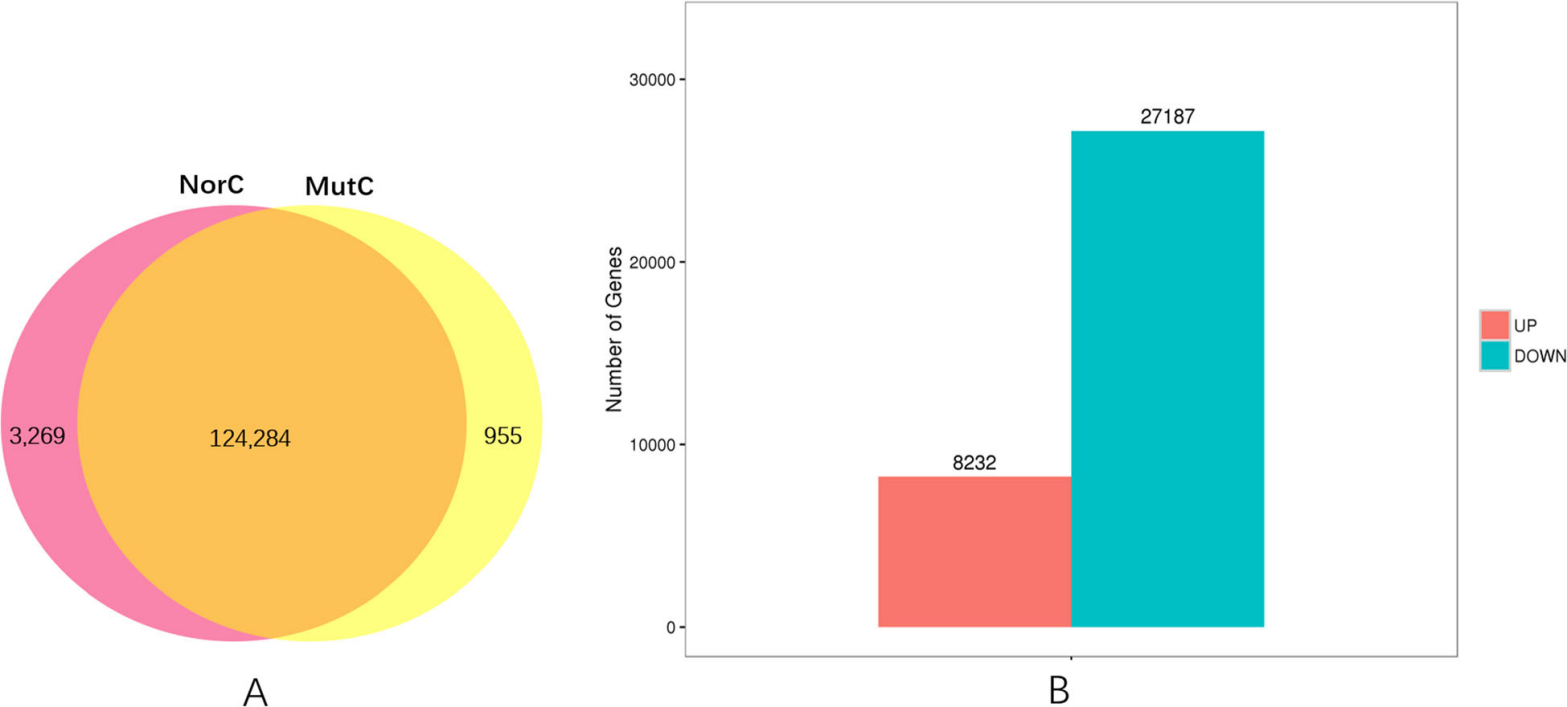
**Venn diagram of the number of expressed genes.** (**a**) Venn diagram of the number of genes expressed in the normal capitula (NorC) and the mutant capitula (MutC). (**b**) Number of up-regulated and down-regulated genes between the normal and mutant capitula

A total of 124,284 unigenes were shared by the normal and mutant capitula (Fig. 5a). In contrast, 3269 and 955 unigenes were specifically expressed in the normal and mutant capitula, respectively.

Genes differentially expressed between mutant and normal capitula.

The transcriptomes of the normal and mutant capitula were compared, and the reads were mapped to the reference transcriptome. A total of 35,419 DEGs (8232 up-regulated and 27,187 down-regulated in the mutant capitula relative to the corresponding levels in the normal capitula) were identified between the normal and mutant capitula (Fig. 5b). The correlation coefficient for the gene expression levels in the normal and mutant capitula was 0.8897, which was determined using an algorithm developed from the correlation scatter plot.

A total of 131 DEGs were specifically expressed in the mutant capitula, including TCP1 and AP2/ERF domain-containing genes. Conversely, 2132 DEGs were specifically expressed in the normal capitula, including some important transcription factor genes (*MYB*, *GRAS*, and *BTF3* genes), ubiquitin-conjugating enzyme genes, zinc finger protein genes, and many unannotated genes. These genes may have important functions in developing chrysanthemum flowers, especially during the pistil determination and development stage. The production of normal capitula composed of ray florets with normally developed pistils and purple corollas and mutant capitula containing ray florets with green corollas and vegetative buds may be due to significant differences in the expression of these genes. Details regarding the annotation of the DEGs specifically expressed in the mutant and normal capitula are provided in Additional files 2 and 3, respectively.

The GO and KEGG pathway enrichment analyses of the DEGs uncovered differences in biological processes and pathways between the mutant and normal capitula. The expression levels of 256 genes annotated with the ‘reproduction’ GO term (GO:0000003) in the biological process category were all considerably lower in the mutant capitula than in the normal capitula. Of these genes, 11 were specifically expressed in the normal capitula, including WD40 and UBA1-like protein-encoding genes. These genes may play important roles in the regulatory pathways related to chrysanthemum reproduction (Additional file 4).

A total of 6733, 7216, and 3879 DEGs were enriched in the biological process, molecular function, and cellular component categories, respectively (Additional files 5–7). In the biological process category, the main terms were ‘metabolic process’ (GO:0008152, 5128 DEGs), ‘cellular process’ (GO:0009987, 4758 DEGs), and ‘single-organism process’ (GO:0044699, 4017 DEGs). In the molecular function category, the most represented terms were ‘catalytic activity’ (GO: GO:0003824, 5765 DEGs), ‘binding’ (GO:0005488, 3903 DEGs), and ‘organic cyclic compound binding’ (GO:0097159, 2386 DEGs). Finally, in the cellular component category, the most common terms were ‘cell’ (GO:0005623, 2633 DEGs), ‘cell part’ (GO:0044464, 2630 DEGs), and ‘intracellular’ (GO:0005622, 2490 DEGs). Thus, the physiological and biochemical activities involved in metabolic, cellular, and single-organism processes differed between the mutant and normal capitula. In total, 16,342 down-regulated and 5485 up-regulated DEGs in the mutant capitula relative to the corresponding levels in the normal capitula were enriched in many KEGG pathways (Additional files 8 and 9). Interestingly, all of the DEGs enriched in the ‘brassinosteroid biosynthesis’ (ko00905) and ‘plant hormone signal transduction’ (ko04075) KEGG pathways were expressed at lower levels in the mutant capitula than in the normal capitula, implying that plant hormone signal transduction activities were suppressed in the mutant capitula. The enriched GO terms and KEGG pathways are listed in Additional files 5–9.

### Important transcription factors differentially expressed between mutant and normal capitula

A total of 3921 important transcription factor genes from 52 classes were detected, among which 963 from the following transcription factor families were substantially differentially expressed between the normal and mutant capitula: AP2 (14 members), ARF (35 members), B3 (41 members), BBR-BPC (3 members), BES1 (8 members), bHLH (75 members), bZIP (22 members), C2H2 (84 members), C3H (51 members), CAMTA (2 members), CO-like (5 members), DBB (11 members), Dof (9 members), E2F/DP (4 members), ERF (98 members), FAR1 (15 members), G2-like (39 members), GATA (8 members), GeBP (1 member), GRAS (29 members), GRF (1 member), HB-other (6 members), HB-PHD (1 member), HD-ZIP (51 members), HSF (20 members), LBD (7 members), LSD (1 member), MIKC (12 members), M-type (9 members), MYB (80 members), NAC (25 members), NF-X1 (12 members), NF-YA (3 members), NF-YB (8 members), NF-YC (5 members), Nin-like (40 members), S1Fa-like (6 members), SBP (13 members), SRS (2 members), TALE (17 members), TCP (3 members), Trihelix (22 members), WRKY (56 members), YABBY (1 member), and ZF-HD (8 members). More specifically, the ERF, C2H2, MYB, bHLH, and WRKY transcription factor families respectively had 98, 84, 80, 75, and 56 members with expression levels that were extremely different between the normal and mutant capitula. Additionally, some important transcription factor genes were expressed only in the normal capitula, including 36 C2H2 genes, 6 bZIP genes, 5 bHLH genes, 5 MYB genes, 4 HB-other genes, 2 C3H genes, 2 E2F/DP genes, 2 GATA genes, 1 ERF gene, 1 HSF gene, 1 NF-X1 gene, 1 TALE gene, and 1 Trihelix gene.

These results suggest that many transcription factors are important for floral development, but the functions of some transcription factors in developing flowers remain to be investigated. The important transcription factor genes substantially differentially expressed between the normal and mutant capitula may be crucial for the phenotypic variations between the normal and mutant capitula. These genes are presented in Additional file 10.

### Identification and expression analysis of genes involved in the photoperiod and GA pathways in chrysanthemum

**Fig. 6:**
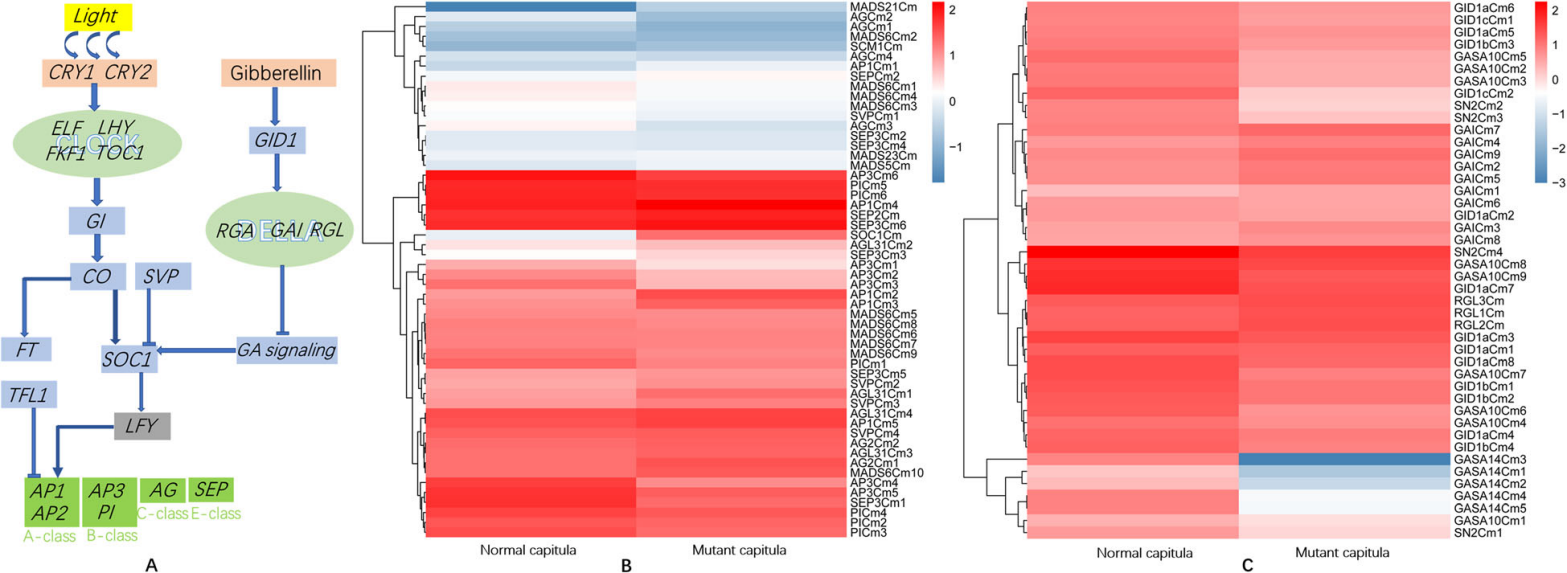
Schematic of the flowering regulatory networks involved in the chrysanthemum photoperiod and GA pathways and the heat maps comparing MADS-box gene expression as well as GA pathway gene expression between normal and mutant capitula. (A) Schematic of the flowering regulatory networks involved in the *Chrysanthemum morifolium* photoperiod and GA pathways. Arrows indicate activation. Bars indicate repression. All homologs of the genes involved in the photoperiod pathway are listed in Additional file 11. (B) Heat maps comparing MADS-box gene expression between normal and mutant capitula in chrysanthemum. Columns and rows in the heat maps represent samples and genes, respectively. Sample names are provided below the heat maps. The color scale indicates gene expression fold-changes. Red and blue respectively reflect high and low expression levels. All homologs of the MADS-box genes are listed in Additional file 12. (C) Heat maps comparing GA pathway gene expression between normal and mutant capitula in chrysanthemum. Columns and rows in the heat maps represent samples and genes, respectively. Sample names are provided below the heat maps. The color scale indicates gene expression fold-changes. Red and blue respectively reflect high and low expression levels. All homologs of the genes involved in the GA pathway are listed in Additional file 13

As a typical short-day plant, chrysanthemum can flower in response to a single short day. Homologs of the important regulators of the photoperiod pathway in chrysanthemum were identified. Molecular genetic studies have identified many genes required for responses to the day length, with some encoding important regulators of flowering, whereas other genes encode components of the light signal transduction pathways or pathways involved in circadian signaling, including PHYTOCHROME (PHY), CRYPTOCHROME (CRY), LATE GIGANTEA (GI), and FKF1 (Flavin binding, Kelch repeat, F-box protein 1). In this study, many genes identified based on the transcriptome sequences were revealed as homologs of photoreceptor and circadian clock components involved in the photoperiod pathway (Fig. 6a). On the basis of the protein annotations of the mutant and normal capitula transcriptome sequences, many genes were identified, including several *CRY1* and *CRY2* homologs as well as homologs of *PHYA*, *PHYB*, *FKF1*, *LHY*, *EFL1*, *EFL3*, *EFL4*, *TOC1*, and *GI*. Moreover, homologs were detected for *CONSTANS* (*CO*), which is critical for the photoperiod response, and for *FT* (*Flowering Locus T*), which is targeted by CO. Many MADS-box genes are important for promoting floral meristem identity, including *SHORT VEGETATIVE PHASE* (*SVP*), *SUPPRESSOR OF CONSTANS1* (*SOC1*), and *APETALA1* (*AP1*). *PISTILLATA* (*PI*) is a floral organ identity gene that specifies petal and stamen identities in the *A. thaliana* flower [20] . Additionally, AGAMOUS (AG) interacts with LEAFY (LFY) and TERMINAL FLOWER1 (TFL1) to maintain the identity of an existing floral meristem [21]. We identified homologs of these MADS-box genes. *APETALA2* (*AP2*) encodes an important promoter of floral meristem identity. Two *AP2* homologs were identified. *LEAFY* (*LFY*), which is vital for the regulation of floral meristem identity, is initially expressed very early throughout the presumptive floral meristem. We did not detect the expression of *LFY* homologs in the mutant and normal ‘ZY’ capitula, probably because these genes were no longer expressed at the full-bloom stage of the capitula. The *SOC1* homolog identified in this study encodes an upstream regulator of *LFY* expression. Interestingly, its expression level was significantly higher in the mutant capitula than in the normal capitula. As an A-class-like gene, *AP1* expression is directly activated by LFY [22, 23]. The *AP1* homologs identified in this study were all more highly expressed in the mutant capitula than in the normal capitula.

Another A-class gene, *AP2*, is not a MADS-box gene and it encodes a transcription factor in the AP2/EREBP family. Two *AP2* homologs were identified, both of which were more highly expressed in the normal capitula than in the mutant capitula. Most core eudicot species include three distinct B-class gene lineages: *PI*, *euAP3*, and *TM6*; however, *TM6*-like genes seem to have been lost in *Arabidopsis* and *Antirrhinum* species [24]. In the current study, we identified *PI* and *AP3* homologs, but the expression of the *TM6*-like genes was undetectable. In contrast, *TM6*-like gene expression was detected in chrysanthemums in earlier studies [25, 26]. We also identified homologs of the C-class gene *AG* and E-like MADS-box genes in this study. The A-, B-, C-, and E-like genes were all expressed in the mutant capitula, which lacked normal stamens and pistils. Details regarding the annotation of the important genes involved in the photoperiod pathway are provided in Additional file 11.

A comparison of the expression of the detected MADS-box genes between the mutant and normal capitula revealed that the expression levels of many *AP1*, *SEP*, and *AGL* homologs were slightly higher in the mutant capitula than in the normal capitula. In contrast, the *AP3* and *PI* homologs were expressed at lower levels in the mutant capitula than in the normal capitula, with *AP3* homolog expression in the mutant capitula less than half of that in the normal capitula (Fig. 6b). This finding may provide researchers with an important clue regarding the molecular mechanism underlying the phenotypic variations between normal and mutant capitula. Details regarding the annotation of the MADS-box genes are provided in Additional file 12.

Previous research proved that GAs, sugars, and light help regulate various pathways required to complete the flower development process [8] . The circadian clock is affected by GA signaling, which is controlled by the transcriptional regulation of two *GAINSENSITIVE DWARF1* (*GID1*) GA receptor genes (*GID1a* and *GID1b*) in *A. thaliana* [27]. Earlier studies demonstrated that GA promotes *A. thaliana* petal, stamen, and anther development by inhibiting the function of the DELLA proteins encoded by *REPRESSOR OF ga1–3* (*RGA*), *GA-INSENSITIVE* (*GAI*), *RGA-LIKE1* (*RGL1*), *RGL2*, and *RGL3*. The *GID1a*, *GID1b*, and *GID1c* genes of *A. thaliana* have been identified [28]. The expression of *GASA* genes is up-regulated by GA and down-regulated by the DELLA proteins GAI and RGA, which are involved in stem elongation or floral development [29]. In this study, we identified homologs of DELLA protein-encoding genes (*RGA*, *GAI*, and *RGL*) as well as *GID1* (*GID1a*, *GID1b*, and *GID1c*), and *GASA* (*GASA10* and *GASA14*) genes. Most of the *RGA*, *GAI*, *RGL1*, *RGL2*, and *RGL3* homolog expression levels were significantly higher in the mutant capitula than in the normal capitula. Additionally, the homologs of GA receptor genes (*GID1a*, *GID1b*, and *GID1c*) and GA-regulated protein-encoding genes (*GASA10* and *GASA14*) were expressed at lower levels in the mutant capitula than in the normal capitula (Fig. 6c). Therefore, the GA signaling pathway was probably suppressed in the mutant capitula. Details regarding the annotation of the important genes involved in the GA pathway are provided in Additional file 13.

### Identification and analysis of important regulatory and functional genes in the anthocyanin biosynthesis pathway and the pigments in the corollas of chrysanthemum florets

The MYB-bHLH-WD40 (MBW) activator complexes modulate the expression of downstream genes required for flavonoid biosynthesis in plants. These complexes are composed of R2R3 MYB transcription factors (MYB), the basic helix-loop-helix (bHLH) transcription factors [e.g., Glabra 3 (GL3), Transparent Testa 8 (TT8), and Enhancer of Glabra3 (EGL3)], and the WD40-repeat protein TRANSPARENT TESTA GLABRA1 (TTG1) [30]. The MBW activator complexes directly mediate the expression of late anthocyanin biosynthetic genes, including chalcone isomerase (*CHI*), flavonoid 3′-hydroxylase (*F3′H*), dihydroflavonol reductase (*DFR*), and anthocyanin synthase (*ANS*) genes, leading to the accumulation of anthocyanins [31].

**Fig. 7:**
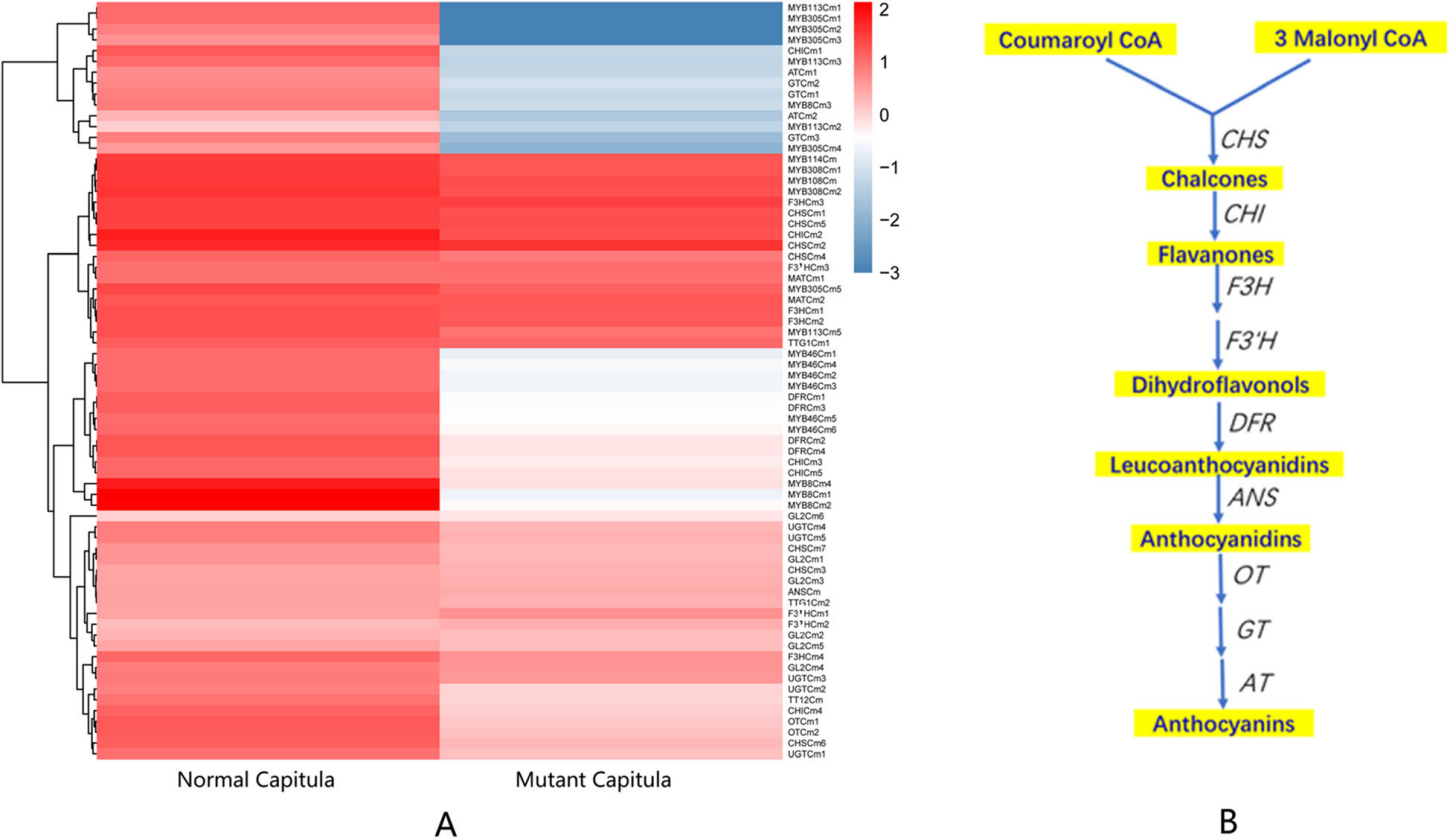
Heat maps comparing anthocyanin biosynthesis pathway gene expression between normal and mutant capitula as well as the anthocyanin biosynthesis pathway and identified regulatory genes. (A) Heat maps comparing anthocyanin biosynthesis pathway gene expression between normal and mutant capitula in chrysanthemum. Columns and rows in the heat maps represent samples and genes, respectively. Sample names are provided below the heat maps. The color scale indicates gene expression fold-changes. Red and blue respectively reflect high and low expression levels. (B) Anthocyanin biosynthesis pathway and the regulatory genes identified based on the chrysanthemum transcriptome. CHS: chalcone synthase, CHI: chalcone isomerase, F3H: flavanone 3-hydroxylase, F3′H: flavonoid 3′-hydroxylase, DFR: dihydroflavonol 4-reductase, ANS: anthocyanidin synthase, OT: 3-O-glucoside-6″-O-malonyltransferase, GT: glucosyltransferase, AT: acyltransferase. The genes in the anthocyanin biosynthesis pathway are listed in Additional file 14

To explore the molecular basis of the flower color differences between the normal and mutant capitula, we identified and analyzed the expression of genes encoding the R2R3 MYB, bHLH, and WD40-repeat proteins, including the homologs of *MYB113*, *MYB114*, *MYB305*, *MYB46*, *Glabra 2* (*GL2*), *Transparent Testa 12* (*TT12*), and *TTG1*. The expression levels of most of the R2R3 MYB genes were significantly down-regulated in the mutant capitula, with some genes not expressed at all (Fig. 7a, Additional file 14). Similarly, the expression levels of the bHLH and WD40-repeat protein genes (*GL2*, *TT12*, and *TTG1*) were also considerably down-regulated in the mutant capitula. Hua Li et al. suggested that MdMYB8 contributes to the regulation of flavonoid biosynthesis, with the overexpression of *MdMYB8* promoting flavonol biosynthesis in crabapple [32]. In this study, four *MYB8*-like genes (*MYB8Cm1*, *MYB8Cm2*, *MYB8Cm3*, and *MYB8Cm4*) were not expressed in the mutant capitula lacking anthocyanins; the lack of expression was confirmed by quantitative real-time PCR (qRT-PCR). Flavonol is an upstream substrate for anthocyanin biosynthesis. Therefore, this result implied these four *MYB8*-like genes may encode important regulators of anthocyanin biosynthesis in the corollas of chrysanthemum florets.

The key functional genes in the anthocyanin biosynthesis pathway, including genes encoding chalcone synthase, chalcone flavonone isomerase, flavanone 3-hydroxylase, flavonoid 3′-hydroxylase, dihydroflavonol 4-reductase, glucosyltransferase, 3-O-glucoside-6″-O-malonyltransferase, acyltransferase, and anthocyanidin synthase, were identified based on the transcriptome (Fig. 7b). Most of the late anthocyanin biosynthetic genes were expressed at lower levels in the mutant capitula than in the normal capitula (Fig. 7a).

Interestingly, all four *DFR* homologs and one *CHI* homolog were expressed at extremely low levels (close to 0) in the mutant capitula. A qRT-PCR analysis indicated that the *CHI* homolog was not expressed in the mutant capitula, but was expressed in the normal capitula. Information regarding the annotation of these genes in the anthocyanin biosynthesis pathway is provided in Additional file 14.

**Fig. 8:**
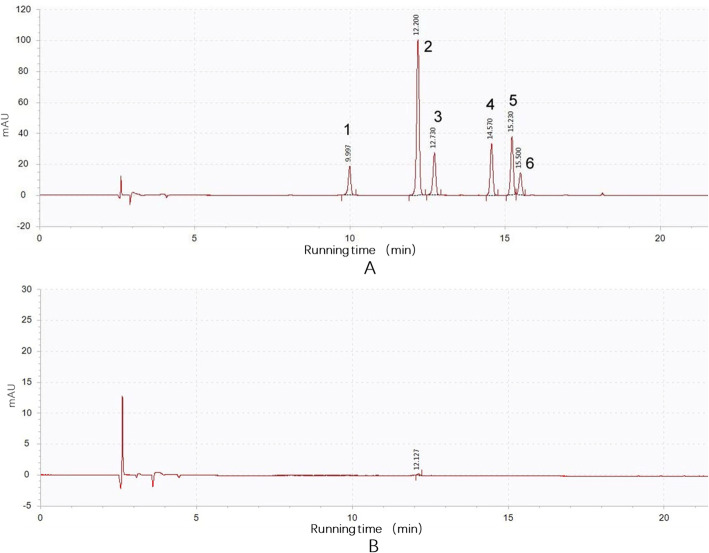
Qualitative analysis of the pigments in the normal (A) and mutant (B) capitula. 1. delphinidin; 2. cyanidin; 3. petunidin; 4. pelargonidin; 5. peonidin; 6. malvidin

The pigment types and contents determine the diversity in flower colors. A qualitative analysis of the pigments in the florets of normal and mutant capitula was performed using an HPLC system. Anthocyanins were detected in the florets of normal capitula, but not in the florets of mutant capitula (Fig. 8b). The detected anthocyanins were mainly delphinidin, cyanidin, petunidin, pelargonidin, peonidin, and malvidin (Fig. 8a). Accordingly, the down-regulated expression of genes encoding MBW activator complex components inhibited the expression of late anthocyanin biosynthetic genes, including *CHI* and *DFR*, leading to an anthocyanin deficiency in the mutant capitula.

### Verification of gene expression profiles in qRT-PCR assays

To further verify the expression profiles of the unigenes revealed following the Illumina sequencing analyses, 16 unigenes were selected for a qRT-PCR analysis of the mutant and normal capitula originally used for the RNA-seq experiment. Four *MYB*-like genes (*MYB8Cm1*, *MYB8Cm2*, *MYB8Cm3*, and *MYB8Cm4*) and one *CHI* gene (*CHICZ-1*) were selected because of their important regulatory effects on anthocyanin biosynthesis. The other analyzed genes were selected randomly, including one *COP1* gene (*COP1CZ*), one *EF2* gene (*EF2CZ*) specifically expressed in the normal capitula, one histone *H2B* gene (*HIS2BCZ*), one catalase gene (*CAT3CZ*), one photosystem II 5 kDa protein gene (*PSBTCZ*), one annexin D8 gene (*ANN2CZ*), one arginine kinase gene (*ARGKCZ*), and four unigenes encoding uncharacterized proteins (*UnknownCZ1*, *UnknownCZ2*, *UnknownCZ3*, and *UnknownCZ4*). The resulting data for all 16 genes were consistent with the sequencing data (Additional file 15, Fig. 9).

**Fig. 9:**
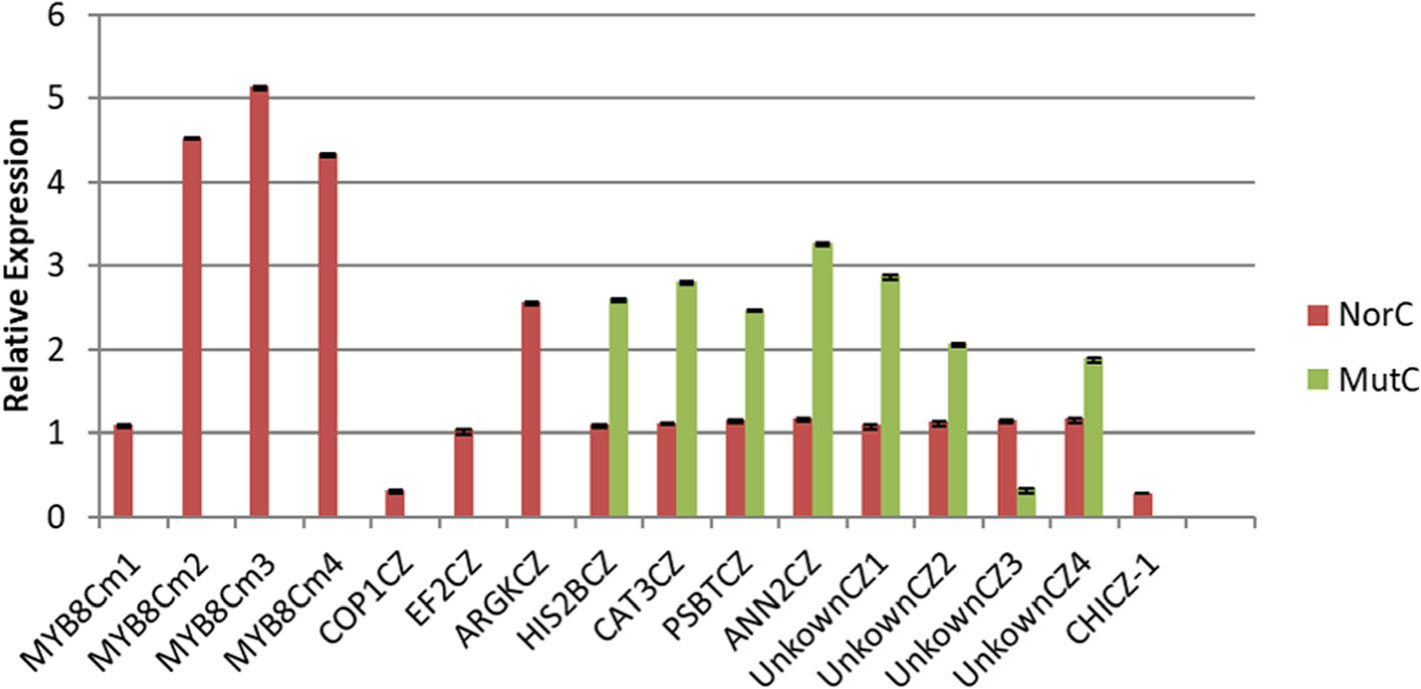
Expression profiles of 16 genes in Chrysanthemum morifolium revealed by qRT-PCR

## Discussion

The normal and mutant capitula differed primarily in the color of ray floret corollas (the normal and mutant capitula were purple and green, respectively) and the replacement of the ray floret pistils of the normal capitula with vegetative buds in the mutant capitula. A whole-transcriptome analysis of the DEGs in the mutant and normal capitula reflected the complexity of the regulatory machinery underlying the phenotypic differences between the capitula. A qualitative analysis of pigments revealed anthocyanins were not synthesized and did not accumulate in the florets of the mutant capitula. A gene expression analysis indicated the down-regulated expression of MBW activator complex genes inhibited the expression of late anthocyanin biosynthetic genes, leading to the deficiency of anthocyanins in the mutant capitula. Furthermore, as one of the major flower pigments in higher plants, the synthesis and accumulation of anthocyanins is an integral part of flower development in most plant species and is tightly linked with petal cell expansion. The activation of the anthocyanin biosynthesis pathway during petal development requires both environmental and endogenous signals, but the endogenous regulatory system is the main factor controlling anthocyanin biosynthesis in developing flowers [8]. Therefore, the anthocyanin deficiency in the mutant capitula is likely related to the mutation causing the ray floret pistils to be replaced by vegetative buds. This mutation may be associated with the differences in the expression of important transcription factor genes and phytohormone signaling pathway genes between the normal and mutant capitula.

### The anthocyanin deficiency in the mutant capitula may be related to a mutation to the ray floret pistils that may be required for anthocyanin biosynthesis in the corollas

In *Petunia hybrida*, GAs, sugars, and light are required for inducing the transcription of anthocyanin biosynthesis genes and the accumulation of pigments in developing corollas [25]. An earlier study proved that in the initial stages of *P. hybrida* flower development, GAs produced by the anthers control anthocyanin biosynthesis and accumulation in the corollas by activating the transcription of specific anthocyanin biosynthesis pathway genes, and that the removal of stamens during the early flower development stage inhibits anthocyanin biosynthesis in the corollas [33]. Tiancong Qi et al. revealed GA and jasmonate coordinately activate the MBW complex by inducing the degradation of DELLA proteins and JASMONATE ZIM-domain proteins [34]. A study by G. W. M. Barendse et al. confirmed that in *P. hybrida* and *Lilium* species, unpollinated styles and ovaries contain GA, with more GA in the ovaries than in the styles [35].

In *C. morifolium*, the capitula of many varieties (e.g., ‘Ping Pong’) have only ray florets because of the extensive breeding for the double-flowered trait. Interestingly, most of the capitula in *C. morifolium* ‘ZY’ comprise ray florets, but no disk florets. In this study, a comparison of the GA signaling pathway gene expression between the normal and mutant capitula indicated that the GA signaling pathway was probably suppressed in the mutant capitula because of a lack of normal pistils, which likely prevented the mutant capitula from synthesizing anthocyanins. Therefore, we hypothesized that when the pistils of ray florets are mutated and replaced by vegetative buds, the anthocyanin biosynthesis in the corollas is blocked. Consequently, normal pistils may be required for the anthocyanin biosynthesis in chrysanthemums.

### Anthocyanin deficiency may also be related to the down-regulated expression of the B-class MADS-box genes *AP3* and *PI* in the mutant capitula

Katsutomo Sasaki et al. revealed the synergistic effects of the proteins encoded by *PI* and *AP3* homologs (*TfGLO* and *TfDEF*) in torenia plants. Anthocyanins accumulate similarly in the sepals and petals of *TfGLO*-overexpressing plants, which produce purple-stained sepals. Plants in which *TfGLO* expression is suppressed have flowers with serrated petals, whereas plants with suppressed *TfDEF* expression produce flowers with partially decolorized petals. Both *TfGLO*- and *TfDEF*-suppressed plants have some sepal-like cells at their petal surfaces [36]. Accordingly, the B-class MADS-box genes *AP3* and *PI* may be involved in anthocyanin biosynthesis. In the current study, we determined that the *AP3* and *PI* homologs were expressed at relatively low levels in the mutant capitula. More specifically, *AP3* homolog expression in the mutant capitula was less than half of that in the normal capitula. Additionally, anthocyanins did not accumulate in the mutant capitula. Therefore, the anthocyanin deficiency of the mutant capitula may be related to the down-regulated expression of the B-class MADS-box genes *AP3* and *PI*.

### Protective mechanisms may be disrupted in mutant inflorescences because of a repressed jasmonate pathway, which prevents the activation of anthocyanin biosynthesis

Plants have developed constitutive and inducible defenses against pests and pathogens. The inducible defenses depend on the combined effects of jasmonate and ethylene [37]. In this study, the expression levels of the DEGs associated with the KEGG pathway ‘plant hormone signal transduction’ (ko04075) were significantly lower in the mutant capitula than in the normal capitula. These DEGs included jasmonate response factor genes and ethylene-responsive sensor genes. Additionally, ‘defense mechanisms’ (673, 0.77%) was one of the smallest groups among the 25 KOG categories. Therefore, the protective mechanisms in mutant inflorescences may be inhibited because of a repressed jasmonate pathway.

Jasmonate up-regulates the expression of several anthocyanin biosynthetic genes essential for anthocyanin accumulation [38]. Zhihong Peng et al. reported that jasmonate-induced anthocyanin accumulation is suppressed by a brassinosteroid deficiency and blocked BR signaling. Brassinosteroids enhance jasmonate-induced anthocyanin accumulation in *A. thaliana* seedlings by regulating the expression of late anthocyanin biosynthetic genes (e.g., *DFR*, *LDOX*, and *UF3GT*) [39]. In the current study, the DEGs associated with the KEGG pathway ‘brassinosteroid biosynthesis’ (ko00905) were expressed at lower levels in the mutant capitula than in the normal capitula, with the expression of some of the DEGs almost undetectable in the mutant capitula. Similarly, the expression levels of four *DFR* homologs were very low in the mutant capitula. Thus, anthocyanin biosynthesis is not activated probably because of the suppressed jasmonate pathway due to the brassinosteroid deficiency.

### The transcription factor genes expressed in the normal capitula, but not in the mutant capitula, may be associated with the mutation to the pistils of ray florets

In this study, 963 of the 3921 detected important transcription factor genes were substantially differentially expressed between the normal and mutant capitula, including members of the ERF, C2H2, MYB, bHLH, and WRKY transcription factor families. Interestingly, some transcription factor genes were expressed exclusively in the normal capitula, including 35 C2H2 genes, 7 MYB genes, 6 bZIP genes, 6 bHLH genes, 4 HB-other genes, 2 C3H genes, 2 E2F/DP genes, 2 GATA genes, 1 ERF gene, 1 HSF gene, 1 NF-X1 gene, 1 TALE gene, and 1 Trihelix gene. Of these genes, the lack of expression of four MYB genes (*MYB8Cm1*, *MYB8Cm2*, *MYB8Cm3*, and *MYB8Cm4*) in the mutant capitula was confirmed by qRT-PCR.

The C2H2 zinc finger transcription factors play important roles in many biological processes related to plant growth and development, hormone signaling, and stress responses [40]. In many plants, the C2H2 zinc finger proteins influence the tolerance to salt, cold, drought, and oxidative stresses as well as responses to light stress and pathogens [40]. Additionally, some members help regulate floral development. For example, SIZF2 affects flower and leaf shapes in *A. thaliana*. Previous investigations proved that the protein encoded by the *SUPERMAN* (*SUP*) gene determines the boundary between the stamen and carpel whorls, suppresses B-class gene expression, and promotes stem cell termination in the fourth whorl of *A. thaliana* flowers [40–42] . In the present study, 35 C2H2 genes were not expressed in the mutant capitula, implying the encoded zinc finger transcription factors may be important for pistil development or the anthocyanin biosynthesis and accumulation in the chrysanthemum ray floret corollas.

The basic leucine zipper (bZIP) transcription factors form one of the largest transcription factor families and are critical for controlling plant development and stress responses [43]. These transcription factors are reportedly involved in various floral developmental processes in plants, including pollen development as well as floral transitions and initiation [44, 45]. In the current study, the deficient expression of six bZIP genes in the mutant capitula suggests that the encoded transcription factors are important regulators of chrysanthemum ray floret development.

The MYB transcription factors, which belong to one of the largest and most diverse transcription factor families in the plant kingdom, are common among eukaryotes and play essential roles in diverse physiological and biochemical processes controlling plant growth and development [46, 47]. The bHLH transcription factors, which possess a highly conserved bHLH domain that includes a basic region and an HLH region, are crucial for plant growth and development, metabolic regulation, and responses to environmental changes. The regulatory functions of bHLH transcription factors related to active secondary metabolism, especially anthocyanin biosynthesis, have been a topic of interest among researchers [48]. We observed that several bHLH and MYB genes were not expressed in the mutant capitula, suggesting these genes are essential for regulating normal ray floret development in chrysanthemums. Accordingly, these genes should be more thoroughly functionally characterized. The other transcription factor genes that were not expressed in the mutant capitula may also encode important regulators of chrysanthemum ray floret growth and development, including the HB genes, two C3H genes, two E2F/DP genes, two GATA genes, one ERF gene, one HSF gene, one NF-X1 gene, one TALE gene, and one Trihelix gene. The mutation resulting in the production of vegetative buds in place of the ray floret pistils may be associated with the lack of expression of these transcription factor genes in the mutant capitula. Consequently, the regulatory functions of the transcription factors in developing chrysanthemum flowers should be explored in detail in the future.

## Conclusions

In this study, a comparative transcriptome analysis revealed significant differences in gene expression and signaling pathways between the mutant and normal capitula. The identified DEGs included important regulators of the phenotypic differences between the normal and mutant capitula. Additionally, the transcription factor genes and the genes associated with the photoperiod and GA pathways, floral organ identity, and the anthocyanin biosynthesis pathway were differentially transcribed between the normal and mutant capitula. A qualitative analysis of the pigments in the florets of normal and mutant capitula revealed anthocyanins were synthesized and accumulated only in the florets of the normal capitula. Therefore, the anthocyanin deficiency in the mutant capitula may be related to the mutation of ray floret pistils and their replacement by vegetative buds. Moreover, pistils may be required for the anthocyanin biosynthesis in the corollas of chrysanthemum ray florets. Furthermore, the transcription factor genes expressed in the normal capitula, but not in the mutant capitula, may also be associated with the mutation to the ray floret pistils. The transcriptome analysis described herein provided valuable information regarding the molecular mechanisms underlying the production of normal ray florets with pistils and purple corollas as well as mutant ray florets with green corollas and vegetative buds in *C. morifolium*. The results of this study may be useful for developing enhanced techniques for studying the regulation of flower shapes and colors and for breeding novel chrysanthemum varieties.

## Methods

### Plant materials and RNA extraction

The normal and mutant capitula used in this study were obtained from a cut-flower chrysanthemum variety (*C. morifolium* ‘ZY’, a hybrid of chrysanthemum varieties) cultivated in a greenhouse under an 8-h light/16-h dark cycle at 23 °C at the Beijing Academy of Agriculture and Forestry Sciences (116.3°E, 39.9°N). After the florets of the capitula were fully formed, about 3–6 normal capitula, 3–6 mutant capitula, 3–4 fully expanded leaves, 1–2 g roots, and 1–2 g stems were collected between 9:00 and 12:00 pm. Three biological replicates were collected for each sample. The collected samples were frozen immediately in liquid N_2_ and stored at − 80 °C. Total RNA was extracted from the frozen samples using the RNeasy Plant Mini Kit (Qiagen, China). The RNA was quantified and the quality was assessed using a NanoDrop ND2000 spectrophotometer (Thermo Scientific).

### Library construction, PacBio sequencing, and data processing

Sequencing libraries were constructed and sequenced with the PacBio Sequel system (PacBio, CA, USA). Briefly, total RNA (1 μg) from each of the five tissues was pooled, after which the mRNA was enriched using oligo (dT) magnetic beads. The enriched mRNA was reverse transcribed into cDNA with the Clontech AMSRTer PCR cDNA Synthesis Kit. After a PCR amplification, the BluePippin Size Selection System was used to produce two bins: 0.5–2 and 2–6 kb. A large-scale PCR was performed and the cDNA products were used to construct SMRTbell template libraries with the SMRTbell Template Prep Kit. The SMRTbell templates were annealed to sequencing primers and bound to polymerase, after which they were sequenced on the PacBio Sequel platform using P6-C4 chemistry with 10-h movies by Gene Denovo Biotechnology Co.

The raw sequencing reads were classified and clustered into consensus transcripts using the PacBio IsoSeq pipeline (https://github.com/PacificBiosciences/IsoSeq_SA3nUP) and the SMRT Analysis software suite (https://www.pacb.com/support/software-downloads/). Briefly, circular consensus sequence (CCS) reads were extracted from the subreads BAM file. The CCS reads were classified into four categories: full-length (FL) non-chimeric, FL chimeric, non-FL, and short reads based on the cDNA primers and poly(A) tail signals. The short reads were discarded. The FL non-chimeric reads were clustered using the Iterative Clustering for Error Correction (ICE) software to generate cluster consensus unigenes. Two strategies were employed to improve the accuracy of the PacBio reads. First, the non-FL reads were used to polish the cluster consensus unigenes with the Quiver software to obtain FL-polished high-quality consensus sequences (accuracy ≥99%). Second, the low-quality unigenes were corrected using the Illumina short reads obtained for the same samples with the LoRDEC tool (version 0.8) [49]. Then, the final transcriptome unigene sequences were filtered by removing the redundant sequences with the CD-HIT (version 4.6.7) software (sequence identity threshold of 0.99).

### Illumina sequencing

The total RNA isolated from the normal and mutant capitula was used for Illumina sequencing on an Illumina HiSeq™ 2000 system (Illumina, San Diego, CA, USA). We purified the poly(A) mRNAs, fragmented them into small pieces, and then synthesized the first- and second-strand cDNAs.

The double-stranded cDNAs were purified and resolved for repairing ends and adding a poly(A) tail. Sequencing adapters were then annealed to the short fragments. Briefly, a cDNA library with average insert sizes of 300–500 bp was created and sequenced using the Illumina HiSeq™ 2000 system to generate 100 bp paired-end reads.

### Basic annotation of unigenes

Unigenes were functionally annotated on the basis of a BLASTX alignment with sequences in the following databases (E-value of 1.00E^− 5^): nr (NCBI), Swiss-Prot (http://www.expasy.ch/sprot), KEGG (http://www.genome.jp/kegg), and KOG (http://www.ncbi.nlm.nih.gov/KOG) databases. The optimal alignment results determined the sequence direction of the unigenes. The Blast2GO program was used for the GO annotation of unigenes [50]. Unigenes with the 20 highest scores and no fewer than 33 high-scoring segment pair hits were selected for the Blast2GO analysis. The unigenes were functionally classified using the WEGO software [51].

### Analysis of chrysanthemum transcriptome sequencing results

The number of reads per kilobase of exon model per million mapped reads (RPKM) following the RNA-seq analysis was used to calculate the expression level of each unigene [52]. The chrysanthemum transcriptome was used as a reference to screen and analyze DEGs. A rigorous algorithm was created based on the method of Audic et al. to screen the DEGs [53]. The false discovery rate (FDR) was used to affirm the *P*-value threshold in multiple tests and analyses [54]. An absolute value of the log_2_ (ratio) ≥ 2 and FDR < 0.05 were applied as the thresholds for determining significant differences in gene expression [55]. Only the DEGs with at least a 2-fold change in expression level were used for the differential gene expression analysis.

### Alternative splicing detection

To analyze alternative splicing events in the unigenes, the coding genome reconstruction tool (Cogent) was employed to partition the transcripts into gene families based on the *k*-mer similarity, after which each family was reconstructed into a coding reference genome using De Bruijn graphs [56]. The alternative splicing events of the unigenes were analyzed using the SUPPA tool [57].

### Gene expression analysis based on qRT-PCR

Total RNA was extracted from the normal and mutant capitula as described above. The total RNA was treated with DNase (Promega, USA) and then used as the template to synthesize cDNA with a reverse transcription system (Tiangen, China). A qRT-PCR analysis was completed using the PikoReal Real-Time PCR system (Thermo Fisher Scientific, Germany). Each reaction was carried out in a total volume of 20 μL, with 2 μL first-strand cDNA as the template. The PCR program was as follows: 95 °C for 30 s; 40 cycles of 95 °C for 5 s and 60 °C for 30 s. The gene-specific qRT-PCR primers listed in Additional file 13 were used to determine the relative expression level of each gene. All samples were analyzed in triplicate and the qRT-PCR experiments were performed with three biological replicates. The relative expression levels were calculated using the 2^−ΔΔCt^ method, with the *C. morifolium* protein phosphatase 2A (*PP2Ac*) gene serving as the reference control [58].

### Qualitative analysis of pigments in chrysanthemum flowers

The anthocyanin profiles in the normal and mutant capitula were analyzed by HPLC. A 1.0–10.0 g sample was ground into a fine powder in liquid N_2_ and homogenized via a sonication at 20 °C for 30 min to produce 50-mL anthocyanin extracts [ethyl alcohol: distilled water: hydrochloric acid (2,1:1, v/v/v)] [59]. The extracts were heated in boiling water for 1 h and then centrifuged at 16,000×g for 10 min at 20 °C. The supernatant was passed through a 0.45 μm reinforced nylon membrane filter and then injected into an X Bridge BEH C18 column (250 mm × 4.6 mm × 5 μm), which was used to separate the anthocyanins and flavonols. The column was maintained at 35 °C and water containing 1% (v/v) formic acid (A) and 1% (v/v) acetonitrile (B) was used as the mobile phase. A gradient elution was applied at a flow rate of 0.8 mL/min with the following conditions: 92% A + 8% B, 0 min; 88% A + 12% B, 2 min; 82% A + 18% B, 5 min; 80% A + 20% B, 10 min; 75% A + 25% B, 12 min; 70% A + 30% B, 15 min; 55% A + 45% B, 18 min; 20% A + 80% B, 20 min; 92% A + 8% B, 22 min; 92% A + 8% B, 30 min. The injection volume was 20 μL and the photodiode array detector was set at 530 nm for anthocyanins [60]. Three biological replicates were analyzed for each sample type.

## Supplementary Information

**Additional file 1 Chrysanthemum flowers.** (A) Capitulum. (B) Disk floret. (C) Ray floret.

**Additional file 2.** The DEGs specifically expressed in the mutant capitula

**Additional file 3.** The DEGs specifically expressed in the normal capitula

**Additional file 4.** The DEGs annotated with the “reproduction” GO term (GO:0000003)

**Additional file 5.** The DEGs enriched in the biological process GO category

**Additional file 6.** The DEGs enriched in the molecular function GO category

**Additional file 7.** The DEGs enriched in the cellular component GO category

**Additional file 8.** The enriched KEGG pathways among the DEGs down-regulated in the mutant capitula

**Additional file 9.** The enriched KEGG pathways among the DEGs up-regulated in the mutant capitula

**Additional file 10.** Important transcription factor genes substantially differentially expressed between the normal and mutant capitula

**Additional file 11.** Important photoperiod pathway genes in chrysanthemum

**Additional file 12.** The MADS-box genes identified in chrysanthemum

**Additional file 13.** The genes involved in the GA pathway in chrysanthemum

**Additional file 14.** Important anthocyanin biosynthesis pathway functional genes in chrysanthemum

**Additional file 15 **Primers used for the quantitative real-time PCR analysis of *Chrysanthemum morifolium*

## Data Availability

All data generated or analysed during this study are included in this published article and its supplementary information files.

## Abbreviations

DEG: Differentially expressed gene
PCR: Polymerase chain reaction
qPCR: Quantitative PCR
