# Supplementary figures and images for: Whole-transcriptome analysis of differentially expressed genes in the mutant and normal capitula of Chrysanthemum morifolium
Source: BMC Genom Data. 2021 Jan 25;22:2. doi: 10.1186/s12863-021-00959-2 (PMC7853313; doi:10.1186/s12863-021-00959-2)

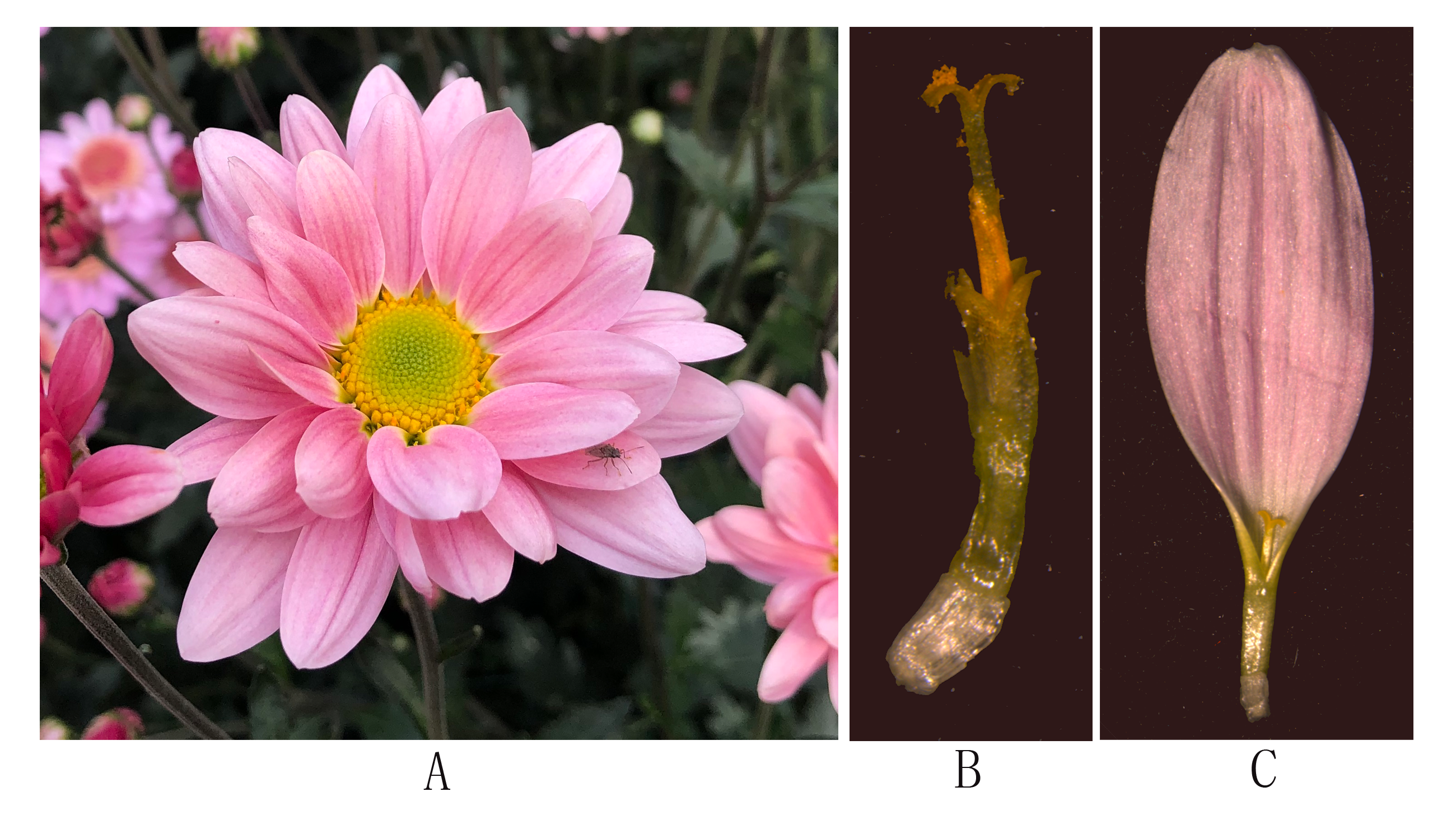

Supplement: Supplementary file 1 — Additional file 1 Chrysanthemum flowers. (A) Capitulum. (B) Disk floret. (C) Ray floret. [file 12863_2021_959_MOESM1_ESM.tif]
