# Supplementary material for: Whole-transcriptome analysis of differentially expressed genes in the mutant and normal capitula of Chrysanthemum morifolium
Source: BMC Genom Data. 2021 Jan 25;22:2. doi: 10.1186/s12863-021-00959-2 (PMC7853313; doi:10.1186/s12863-021-00959-2)
Supplement: Supplementary file 15 — Additional file 15 Primers used for the quantitative real-time PCR analysis of Chrysanthemum morifolium [file 12863_2021_959_MOESM15_ESM.docx]

Table S12 Primers used in real-time quantitative PCR of *Chrysanthemum morifolium*

| Gene name | Forward primer sequence (5′-3′) | Reverse primer sequence (5′-3′) | Correlation between RNA-Seq and qRT-PCR (R2) |
| --- | --- | --- | --- |
| *MYB8CZ-1* | TTCGGCATTGGTCAGGATA | CTTAGCCTCAGGAGTTTCAG | 1 |
| *MYB8CZ-2* | TTCACCGAGCAGTAAAGGA | CGTAAGGACCACCACATTG | 1 |
| *MYB8CZ-3* | CCCTTCTCACATCTGGTCGT | TGGACGATGGAAGAAGACT | 1 |
| *MYB8CZ-4* | ACTGCATTGACCACAGATCC | TCACCCGTGCGACGATACTA | 1 |
| *COP1CZ* | TGTATTCCCGAACTACCTCC | CAGGGAGTGTAAGATTTCGA | 1 |
| *EF2CZ* | TACGGCTACGCTACCTCCAA | GCGATGTAGTTGACGGTGTA | 1 |
| *ARGKCZ* | CAGCCCGCACTATCTGAAT | ATGCGGTCAAAGTTCTGGT | 1 |
| *HIS2BCZ* | GGGTGGTTTGATGTAATG | CAATAACACCACCACTACAT | 0.99 |
| *CAT3CZ* | ATGCTTCAAGGTCGTATC | AGTAATCAACCTCCTCGT | 0.98 |
| *PSBTCZ* | GCAGCAGAGGATGAGGAG | AGCGAAAGAGCACAGATT | 0.99 |
| *ANN2CZ* | TTACATTACTAAGAGCGACCAT | GCCTTCTCAAGAGGGATA | 0.99 |
| *UnkownCZ1* | TTTGCTCTGGCTCCTTT | AGAAAGCATACACCCTAAT | 0.99 |
| *UnkownCZ2* | ACGGAACCACTTACTACAA | TTCCTTCCAGAATGCCTC | 0.99 |
| *UnkownCZ3* | CGGACTGGAGTAGCAAA | TTACCAGTCAACTTAGCC | 0.98 |
| *UnkownCZ4* | CAGCGGCTTAGTGGAATA | CCAAATGTAATGGCGTAA | 1 |
| *PP2Acs* （Reference gene） | ATCAGAACAGGAGGTCAGGG | TAATTTGTATCGGGGCACTT |  |
